# Health-Related Quality of Life among School Children with Parasitic Infections: Findings from a National Cross-Sectional Survey in Côte d'Ivoire

**DOI:** 10.1371/journal.pntd.0003287

**Published:** 2014-12-04

**Authors:** Eveline Hürlimann, Clarisse A. Houngbedji, Richard B. Yapi, Prisca B. Ndri, Kigbafori D. Silué, Gotianwa Soro, Ferdinand N. Kouamé, Thomas Fürst, Jürg Utzinger, Eliézer K. N'Goran, Giovanna Raso

**Affiliations:** 1 Department of Epidemiology and Public Health, Swiss Tropical and Public Health Institute, Basel, Switzerland; 2 University of Basel, Basel, Switzerland; 3 Département Environnement et Santé, Centre Suisse de Recherches Scientifiques en Côte d'Ivoire, Abidjan, Côte d'Ivoire; 4 Unité de Formation et de Recherche Sciences de la Nature, Université Nangui Abrogoua, Abidjan, Côte d'Ivoire; 5 Unité de Formation et de Recherche Biosciences, Université Félix Houphouët-Boigny, Abidjan, Côte d'Ivoire; 6 Programme National de Santé Scolaire et Universitaire, Abidjan, Côte d'Ivoire; 7 Centre for Health Policy, Imperial College London, London, United Kingdom; 8 Department of Infectious Disease Epidemiology, Imperial College London, London, United Kingdom; Case Western Reserve University, United States of America

## Abstract

**Background:**

Parasitic infections are still of considerable public health relevance, notably among children in low- and middle-income countries. Measures to assess the magnitude of ill-health in infected individuals, however, are debated and patient-based proxies through generic health-related quality of life (HrQoL) instruments are among the proposed strategies. Disability estimates based on HrQoL are still scarce and conflicting, and hence, there is a need to strengthen the current evidence-base.

**Methodology:**

Between November 2011 and February 2012, a national school-based cross-sectional survey was conducted in Côte d'Ivoire. Children underwent parasitological and clinical examination to assess infection status with *Plasmodium* and helminth species and clinical parameters, and responded to a questionnaire interview incorporating sociodemographic characteristics, self-reported morbidity, and HrQoL. Validity analysis of the HrQoL instrument was performed, assessing floor and ceiling effects, internal consistency, and correlation with morbidity scores. Multivariate regression models were applied to identify significant associations between HrQoL and children's parasitic infection and clinical status.

**Principal Findings:**

Parasitological examination of 4,848 children aged 5–16 years revealed *Plasmodium* spp., hookworm, *Schistosoma haematobium*, *Schistosoma mansoni*, *Ascaris lumbricoides*, and *Trichuris trichiura* prevalences of 75.0%, 17.2%, 5.7%, 3.7%, 1.8%, and 1.3%, respectively. Anemic children showed a significant 1-point reduction in self-rated HrQoL on a scale from 0 to 100, whereas no significant negative association between HrQoL and parasite infection was observed. The 12-item HrQoL questionnaire proofed useful, as floor and ceiling effects were negligible, internally consistent (Cronbach's alpha = 0.71), and valid, as revealed by significant negative correlations and associations with children's self-reported and clinically assessed morbidity.

**Conclusions/Significance:**

Our results suggest that HrQoL tools are not sufficiently sensitive to assess subtle morbidities due to parasitic infection in Ivorian school-aged children. However, more advanced morbid sequelae (e.g., anemia), were measurable by the instrument's health construct. Further investigations on health impacts of parasitic infection among school-aged children and refinement of generic HrQoL questionnaires are warranted.

## Author Summary

Infectious diseases like malaria and parasitic worms affect hundreds of millions of people, and impact physical and cognitive development of children in Africa, Asia, and the Americas. Over the past 20 years, it was debated how the magnitude of ill-health due to these conditions should be assessed. One proposed strategy was to include patient-based ratings of wellbeing by administration of health-related quality of life (HrQoL) questionnaires. In order to provide new evidence on disability from parasitic infections, we conducted HrQoL interviews with children aged 5–16 years from 92 schools across Côte d'Ivoire. Children were examined for parasitic infections and clinical signs like anemia, malnutrition, and organ enlargement. We compared the self-rated HrQoL of infected and non-infected children and also considered their sociodemographic background. We could not identify lowered HrQoL in infected children, but we found that children with anemia reported a 1-point lower score on a 100-point HrQoL scale in comparison with their non-anemic counterparts. We consider our HrQoL questionnaire as useful and valid, but would recommend its further testing and development in few purposefully selected settings. Further investigation of disability induced by malaria and parasitic worm infections is warranted.

## Introduction

Malaria and the neglected tropical diseases (NTDs) are still of considerable public health relevance in the tropics and subtropics and their successful control is a key issue toward progress of the millennium development goals (MDGs) and the post-2015 agenda of sustainable development [1]–[4]. Preschool-aged children are considered at highest risk of malaria, whereas school-aged children are the most affected by parasitic worm infections (helminthiases) [5]–[7]. The assessment of the precise burden attributable to parasitic infections, however, is a difficult issue and there is ongoing discussion and debate [8], [9]. Over the past 20 years, the magnitude of health loss due to diseases, injuries, and risk factors has been increasingly expressed in disability-adjusted life years (DALYs). This metric is a combined measure of premature death and years of life lived with disability. For measuring the burden of helminthiases and other NTDs, specific disability weights (DWs) of morbid sequelae are considered and, by convention, scaled on an axis from 0 (no health loss) to 1 (health loss equivalent to death) [10]. Former estimates were often criticized for underestimating the true burden of infectious diseases, due to separating out morbidity (e.g., anemia), although such morbidity is partially associated with infection (e.g., hookworm and *Plasmodium*). Additionally, cultural and socioeconomic contexts are insufficiently taken into account, and DWs were usually based on expert opinion; thus, ignoring community- or patient-based appraisal [11]–[13]. Meanwhile, the Global Burden of Disease (GBD) consortium presented estimates for the year 2010 by incorporating different sequelae to capture direct consequences of infections and judgments on health losses from the general public in culturally and socioeconomic diverse settings [14], [15]. Nonetheless, the use of generic health status measurement instruments to expand the GBD approach has been discussed by the lead authors of the GBD 2010 study [15], thus partially addressing concerns that have been articulated a decade ago [16], [17].

The discussed generic health status measurement instruments evaluate health burden in a comprehensive way based on health-related quality of life (HrQoL) and typically include domains on physical, mental, and social wellbeing, and a visual analogue scale (VAS) for subjective health rating [18]–[20]. Thus far only few studies have assessed HrQoL and derived DWs in individuals with parasitic diseases, indicating the early stage of this approach in the field of parasitology. This issue is further underscored by conflicting results; while negative associations between HrQoL measures and *Trichuris trichiura*, *Schistosoma mansoni*, *Schistosoma haematobium*, and advanced *Schistosoma japonicum* infections were observed [21]–[23], other studies failed to show significant differences in HrQoL and DWs between infected children and their non-infected counterparts [24]–[26]. A weaker explanatory power in previous studies may partly be explained by a lack of cross-cultural validity of the questionnaires. HrQoL instruments have been developed and broadly validated in Europe and the United States of America and were originally designed for adult respondents. Child-friendly versions meanwhile exist [27], [28], but application in different cultural settings imply careful adaptations in language and scoring, thorough pre-testing, and validity analysis.

Considering the scarcity of empirical data on HrQoL assessments in school-aged children with single and multiple species infections, the aim of the present study is to strengthen the current evidence-base of disability due to parasitic diseases among pupils in Côte d'Ivoire. Hence, a cross-sectional school-based survey was carried out using standardized, quality-controlled parasitological and questionnaire tools. Furthermore, we discuss the utility and validity of a HrQoL questionnaire tailored to a given setting with basic elements from readily available tools.

## Methods

### Ethics Statement

The study protocol was approved by the institutional research commissions of the Swiss Tropical and Public Health Institute (Basel, Switzerland) and the Centre Suisse de Recherches Scientifiques en Côte d'Ivoire (Abidjan, Côte d'Ivoire). Ethical approval was obtained from the ethics committees in Basel (EKBB; reference no. 30/11) and Côte d'Ivoire (CNER; reference no. 09-2011/MSHP/CNER-P). Additionally, permission to carry out the study was sought from the Ministry of National Education in Côte d'Ivoire. Directors and teachers of the selected schools, district and local health and education authorities were informed about the purpose and procedures of the study. Written informed consent was obtained from parents and legal guardians of children, whilst children assented orally. Participation was voluntary, and hence, children could withdraw from the study at any time without further obligations. All collected data were coded and kept confidential. Participating children benefited from free of charge deworming with albendazole (single oral dose of 400 mg). Children identified to harbor *Schistosoma* spp. were given praziquantel (single oral dose of 40 mg/kg). In schools where the prevalence of *Schistosoma* infection was above 25%, the entire study sample was treated with praziquantel. Symptomatic malaria cases, defined as having a positive rapid diagnostic test (RDT) and fever, were offered artemisinin-based combination therapy (ACT; using artesunate-amodiaquine) and paracetamol against fever.

### Study Design and Subjects

Between November 2011 and February 2012 (i.e., dry season) we conducted a national cross-sectional, school-based study, including parasitological and clinical examinations, and administered a questionnaire. Our aim was to select approximately 100 schools across Côte d'Ivoire, which we considered as a maximum number of locations that we would be able to visit within a 3-month period and our financial and human resources would allow to cover. A lattice plus close pairs design [29], [30] was applied for the sampling of the schools. In brief, a grid indicating latitude and longitude at a unit of 0.5° was overlaid on a map of Côte d'Ivoire that divides the country into two major ecological zones [31]. The southern ecozone is characterized by abundant rainfall (>1,000 mm per annum) and dense forest vegetation cover, whereas the northern ecozone corresponds to a savannah-type profile with markedly less precipitation. In order to achieve a representative sample of the country, 58 and 42 possible survey locations were retained after randomly drawing from each or every second grid cell of ecozones 1 and 2, respectively, taking into account population density from the last available census in 1998. About 27% of the population was estimated to live in the major urban centers in 2007 [32]. We aimed at including at least one fifth of all schools from urban areas. In total, 94 schools were selected and we double-checked that the schools comprised a minimum of 60 children attending grades 3 and 4, using a recent school inventory from a national UNICEF education program (UNICEF 2010; personal communication). Children attending grades 3 and 4 were considered as capable to express themselves and give reliable answers to questionnaire items on household assets, experienced symptoms and diseases, and HrQoL and may be retrievable in case of followed-up studies. The sample size per school was delimited to 60 children due to financial and operational constraints, considering the high number of schools to be surveyed and the maximum number of children that a survey team could sample in a single day, including questionnaire interviews and detailed laboratory work-up of blood, stool, and urine specimens. This sample size exceeds the minimum of 50 children to be surveyed in a school, as recommended by the World Health Organization (WHO) for collection of baseline information on helminth prevalence and intensity in the school-aged population within large-scale surveys [7].

**Figure 1:**
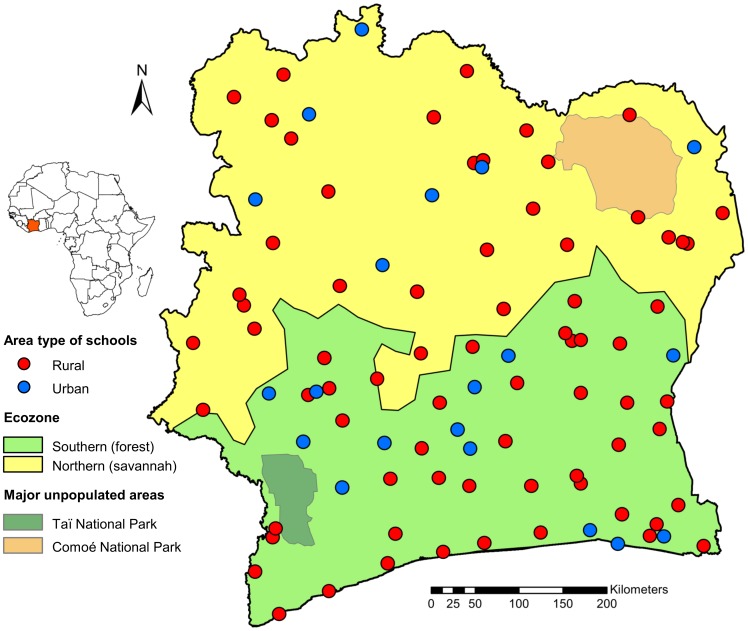
Map of Côte d'Ivoire showing the included schools (n = 92), stratified by rural and urban residential area. The study was conducted between November 2011 and February 2012 among school children aged 5–16 years. The majority (60%) of the enrolled schools were situated in the more densely populated southern ecozone.

Two schools were omitted in the final analysis. One school refused to participate, while another school was subjected to recent deworming. The latter would have biased the results, since signs and symptoms due to chronic helminth infections and HrQoL are likely to change after anthelmintic treatment. The remaining 92 schools are mapped by ecozone, and stratified by rural and urban setting characteristics (Figure 1).

### Field and Laboratory Procedures

In advance of the study conduct, directors and teachers of the selected schools were contacted and they were invited to inform parents or legal guardians of 60 children attending grades 3 and 4. Whenever necessary, children from grade 5 were invited to complement sampling to reach the targeted number of 60 children. Children whose parents/guardians had provided written informed consent were invited for participation. The objectives and procedures of the study were explained on the day of the visit. Children were then asked to provide fresh urine and stool samples in plastic containers distributed upon arrival at school. Additionally, a finger-prick blood sample was taken for preparation of an RDT of malaria (ICT ML01 malaria Pf kit; ICT Diagnostics, Cape Town, South Africa) and thick and thin blood films on microscope slides for subsequent analysis of *Plasmodium* infection. All biological samples were transferred to nearby laboratories and processed the same day. In brief, urine reagent strips (Hemastix; Siemens Healthcare Diagnostics GmbH, Eschborn, Germany) were used to assess microhematuria in urine samples, as a proxy for *S. haematobium* infection [33]. Of note, reagent strips show a high specificity for indirect diagnosis of *S. haematobium* among school-aged children in endemic areas [34]. Duplicate Kato-Katz thick smears [35], using 41.7 mg templates, were prepared from each stool sample. Kato-Katz thick smears were allowed to clear for 30–45 min prior to microscopic examination by experienced laboratory technicians. The number of helminth eggs was counted and recorded for each species separately (i.e., *S. mansoni*, *A. lumbricoides*, *T. trichiura*, hookworm, and other helminths). Blood films were stained with a 10% Giemsa solution and examined under a microscope for *Plasmodium* species identification and quantification of parasitemia (parasites/µl of blood) [36]. For quality control, 10% of the Kato-Katz thick smears and stained blood film slides were re-examined by a senior microscopist. In case of discrepancies (e.g., positive *versus* negative results or counts of parasitic elements differing by more than 10%), slides were read by a third technician and findings discussed until agreement was achieved.

All participating children underwent a clinical examination, conducted by experienced medical staff, which included hemoglobin (Hb) measurement using a HemoCue analyser (Hemocue Hb 301 system; Angelholm, Sweden) to assess anemia, palpation for liver and spleen enlargement, and measurement of body temperature using an ear thermometer (Braun ThermoScan IRT 4520; Kronberg, Germany) for identification of fever cases (≥38.0°C). Two anthropometric measurements were taken (i.e., height in cm and body weight in kg, precision 0.5 kg) for subsequent calculation of children's nutritional status.

### Questionnaire Study

A questionnaire assessing the socioeconomic status, self-reported symptoms and diseases, and HrQoL was administered to all children. Questions on household asset ownership, diseases, and disease-related symptoms were adapted from an instrument previously used in school-based surveys conducted in Côte d'Ivoire [37]. Children were asked for 11 different symptoms (i.e., abdominal pain, blood in stool, blood in urine, diarrhea, dysentery, fatigue, fever, headache, loss of appetite, respiratory problems, and vomiting/nausea) and eight diseases (i.e., cold, cough, eye disease, malaria, malnutrition, schistosomiasis, skin disease, and worms) using a recall period of 2 weeks. To evaluate self-rated HrQoL, the French version of the WHOQOL-BREF tool [18] served as template. Specific questions were dropped and some questions were slightly rephrased to be more specific for the current context, interviewing school-aged children in Côte d'Ivoire. In addition to specific questions focusing on HrQoL, children were asked to rate their general health status using an adapted VAS [38]. This single-item measure basically consists of a thermometer-like scale, in which the anchors are ‘best imaginable health’ and ‘worst imaginable health’, in our case defined as a maximum and minimum value of 10 and 0, respectively. The complete questionnaire instrument was further refined in several rounds of pre-testing in a primary school that was not otherwise involved in the current study. In this pre-testing, children attending grades 2–5 with different cultural backgrounds were included. We determined interview duration using a stopwatch and comprehensibility and appropriateness of the HrQoL part, which was not yet validated from earlier studies, with the goal to achieve a compact, understandable, and locally valid instrument. Questionnaire interviews in the field were conducted by members of the study team and teachers from the selected schools, who were trained beforehand.

### Statistical Analysis

Data were double-entered and cross-checked using EpiInfo version 3.5.3 (Centers for Disease Control and Prevention; Atlanta, United States of America) and analyzed in Stata version 10.1 (Stata Corp.; College Station, United States of America). Only data from children with written informed consent, completed questionnaire, valid parasitological results, and clinical assessments were considered for further analysis.

Socioeconomic data were utilized to calculate a wealth index following an asset-based approach as adopted and explained elsewhere [37], [39]. According to their index score, children were stratified into five economic groups according to wealth quintiles (i.e., most poor, very poor, poor, less poor, and least poor). Data on helminth infections were classified into light, moderate, and heavy, following WHO guidelines [7]. Anemia was defined as having a Hb level below 115 g/l in children aged 5–11 years and 120 g/l in children aged12–15 years [40]. The presence of organ enlargement was defined as having a palpable liver or spleen; the latter of grade 1 or higher using a Hackett's scale [41]. Indicators for malnutrition were calculated according to WHO child growth standards for children aged 5–19 years [42]. They included stunting (height-for-age), wasting (body mass index (BMI)-for-age), and underweight (weight-for-age). The latter is considered a valid measure for nutritional status in children up to 10 years only and was incorporated in a summary measure for malnutrition, defined as Z-score <−2 for any of the three nutritional indicators.

HrQoL questionnaire answers were coded as 1, 2, or 3 (in question 1 up to five codes; Appendix S1) with higher scores indicating fewer problems for a certain issue or activity. HrQoL questionnaire scores were summarized into three main domains on (i) physical, (ii) psychosocial, and (iii) environmental wellbeing. The first comprised the sum of scores from questions 2–6, the second from questions 7–9, and the third from questions 10–12. Each child's overall score on HrQoL was built by summing up individual scores from questions 1–12. Domain and overall raw scores were further converted to a 100-point scale (formula: [(raw score−lowest possible score)/raw score range]×100) [43]. Cronbach's alpha coefficient was used to assess for internal consistency of the HrQoL scores. Overall HrQoL, domain, and VAS scores were subjected to analysis on floor and ceiling effects. Floor or ceiling effects (>15% of respondents achieved lowest or highest possible score) can indicate limited content validity and reduced reliability, whilst responsiveness may be limited since changes in respondents with lowest or highest possible scores cannot be measured [44]. The validity of the HrQoL instrument was further evaluated by assessing relationships of domain, overall HrQoL and VAS scores with symptoms reporting and clinical signs using Spearman rank correlation and linear regression analysis, as appropriate. In order to relate the questionnaire measures with self-reported and clinically assessed morbidity, additional summary variables providing the total number of self-reported symptoms and diseases (n = 19) and clinical signs (n = 7) for each child was generated, with possible ranges of 0 to 19 and 0 to 7, respectively.

Chi square (χ^2^), Fisher's exact, Student-*t*, Kruskal-Wallis, and Wilcoxon rank sum tests were applied, as appropriate, to investigate significant univariate differences between groups for sociodemographic, parasitological, clinical, and HrQoL indicators. Associations between the HrQoL outcome and parasitic infection, infection intensity, and clinical status were assessed using multivariate linear regression analysis with random effects to account for clustering within schools. In case of censored data, we additionally applied tobit regression models. Particular emphasis was placed on total HrQoL and physical wellbeing domain scores as outcome in order to make explicit the physical and non-physical impacts of the health conditions assessed. Explanatories of regression models included sociodemographic, parasitological, and clinical variables. The final models were built, following a stepwise backward elimination approach. Covariates were excluded from the model at a significance level of 0.20 or higher. Relationships between the outcome and remaining explanatory variables were expressed as adjusted mean differences with corresponding 95% confidence intervals (CIs).

## Results

### Operational Results

**Figure 2:**
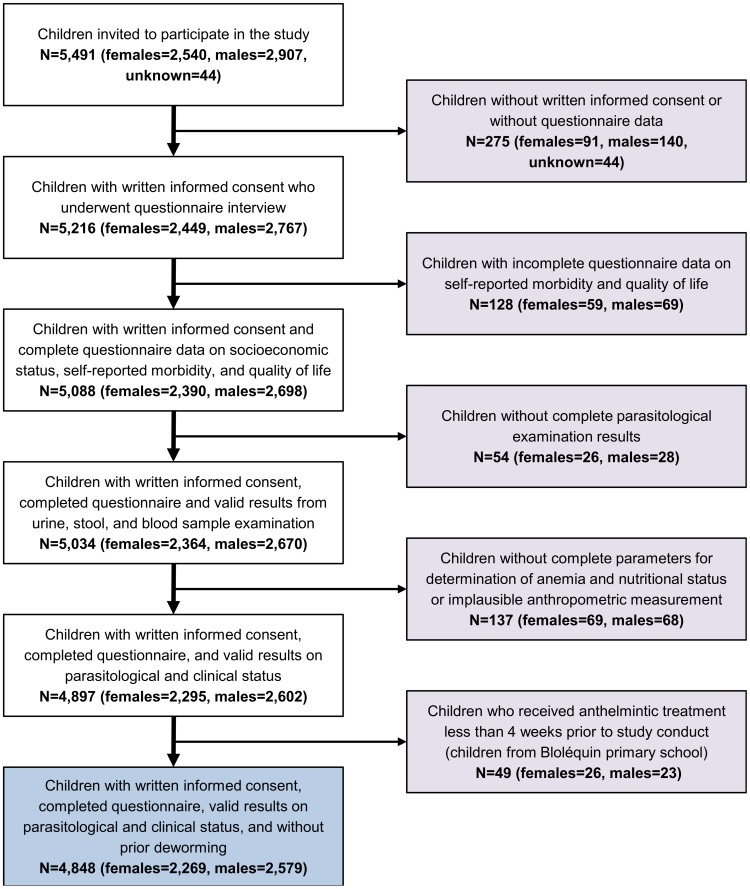
Flow chart, detailing study participation and compliance. The cross-sectional, school-based, national survey was carried between November 2011 and February 2012 in Côte d'Ivoire.

A total of 94 schools across Côte d'Ivoire were visited during the study and 5,491 children invited to participate. Figure 2 depicts the study compliance and participation in the various assessments undertaken. The final sample used for in-depth analysis consisted of 4,848 children from 92 schools with a mean age of 9.8 years (range: 5 to 16 years). These children had complete questionnaire, parasitological, and clinical data and had not received deworming drugs within the past 4 weeks prior to the survey. There were slightly more boys than girls (2,579 *versus* 2,269). 72 schools were considered rural, whilst the remaining 20 (21.7%) were based in urban settings. 4,101 (84.6%) of the children belonged to the two targeted school grades, 3 and 4.

The data set is provided as supplementary information (Data set S1).

### Parasite Infection and Clinical Status

Table 1 summarizes overall prevalence and intensity of parasitic infections, clinical signs, and self-reported symptoms and diseases. Overall 3,635 of the 4,848 children (75.0%) harbored any malaria parasite. *P. falciparum* was the predominant species (74.1%), followed by *P. malariae* (3.9%) and *P. ovale* (0.3%). The latter two *Plasmodium* species occurred mainly as co-infections with *P. falciparum*. Helminth infections; namely, hookworm, *S. mansoni*, *A. lumbricoides*, and *T. trichiura* were observed in 17.2%, 3.7%, 1.8%, and 1.3% of the children, respectively. Microhematuria was found in 5.7% of the children. The majority (95.6%) of soil-transmitted helminth infections were of light intensity, whereas about half of the *S. mansoni*-infected children had moderate- to heavy-intensity infections (≥100 eggs per gram of stool). More than a fourth of all children were found to be anemic (28.7%) or malnourished (28.4%) and a mean number of 6.1 experienced symptoms or diseases were reported.

**Table 1:** Prevalence and intensity of parasitic infections, clinical signs and self-reported symptoms and diseases among 4,848 school children in Côte d'Ivoire.

Parasitic infection	N	%	Morbidity	N	%
*P. falciparum*	3,593	74.1	Observed clinical signs		
*P. malariae*	190	3.9	Anemia^b^	1,391	28.7
*P. ovale*	13	0.3	Any form of malnutrition^d^	1,375	28.4
*Plasmodium* spp.	3,635	75	Stunting^c^	875	18.1
Parasitemia ≥1,000 parasites/µl of blood	1,134	23.4	Wasting^c^	574	11.8
*S. haematobium*	276	5.7	Spleen enlargement^e^	559	11.5
*S. mansoni*	177	3.7	Liver enlargement^e^	126	2.6
Light infection^a^	85	48	Fever (≥38°C)	90	1.9
Moderate infection^a^	60	33.9	Clinical malaria^f^	69	1.4
Heavy infection^a^	32	18.1	Self-reported symptoms		
Hookworm	835	17.2	Headache	2,633	54.3
Light infection^a^	808	96.8	Abdominal pain	2,477	51.1
Moderate infection^a^	16	1.9	Fatigue	2,356	48.6
Heavy infection^a^	11	1.3	Fever	2,335	48.2
*A. lumbricoides*	89	1.8	Vomiting/nausea	1,696	35.0
Light infection^a^	75	84.3	Diarrhea	1,525	31.5
Moderate infection^a^	14	15.7	Blood in stool	1,452	30.0
Heavy infection^a^	0	0	Loss of appetite	1,399	28.9
*T. trichiura*	61	1.3	Respiratory problems	1,301	26.8
Light infection^a^	61	100	Dysentery	1,170	24.1
Moderate infection^a^	0	0	Blood in urine	491	10.1
Heavy infection^a^	0	0	Self-reported diseases		
Soil-transmitted helminths	926	19.1	Cough	2,777	57.3
Light infection^a^	885	95.6	Cold	2,237	46.1
Moderate infection^a^	30	3.2	Malaria	1,472	30.4
Heavy infection^a^	11	1.2	Malnutrition	1,038	21.4
			Eye disease	928	19.1
			Worms	812	16.8
			Schistosomiasis	686	14.2
			Skin disease	635	13.1

Parasite prevalences are provided in % of all included school children. Data on infection intensities are provided as % of all positive cases. Prevalences of clinical or self-reported morbidities are given in % of all included school children.

a Intensities of intestinal helminth infections are categorized according to WHO guidelines [7].

b Defined as hemoglobin levels below 115 g/l and below 120 g/l in children aged 5–11 years and 12–16 years, respectively.

c Calculated according to WHO child growth standards [42]; defined as BMI-for-age (wasting) and height-for-age (stunting) resulting in a Z-score <−2.

d Defined as any of the assessed nutritional indicators resulting in a Z-score <−2; this includes wasting, stunting, and weight-for-age (underweight).

e Defined as palpable liver and spleen (≥grade 1 by Hackett's classification), respectively.

f Clinical malaria is defined as being *Plasmodium*-positive and having fever (≥38°C).

Detailed information on parasitic infections and clinically assessed and self-reported morbidity stratified by sex, age group, residential area, and ecozone are provided in Supporting Information Tables S2 and S3. Boys showed significantly higher infection rates for *P. falciparum*, hookworm, and *S. mansoni* (Table S1). Prevalence rates differed between age groups; while *P. malariae* was more often found in younger children, infections with *Schistosoma* and soil-transmitted helminths were more prevalent in children aged 11–16 years than in their younger counterparts. *Plasmodium* spp. and soil-transmitted helminth infections were most prevalent among the poorest and rural households (all p<0.001). *Plasmodium* spp. was more common in children living in the northern ecozone. Clinical morbidity, such as anemia and indicators for malnutrition, was more pronounced in boys than girls and in older children compared to their younger counterparts (Table S2). Splenomegaly was found to be more common in the younger age group (p = 0.014) and in children from rural and northern settings compared to children living in urban and southern environments (both p<0.001). Anemia (p = 0.049), splenomegaly (p<0.001) and stunting (p<0.001) were significantly lower in children from wealthier households. Furthermore, helminth (OR = 1.69, p<0.001) and *Plasmodium* (OR = 1.44, p<0.05) mono-infected as well as co-infected (OR = 2.0, p<0.001) children showed significantly higher odds ratios (ORs) for anemia than their non-infected peers in multivariable logistic regression analysis. Symptom and disease reporting was higher in girls compared to boys, in older compared to younger individuals, in children from northern regions compared to their counterparts living in the southern ecozone (all p<0.001), and in children from poorer households (p = 0.025).

### Validity of HrQoL Instrument

Table 2 shows the results from the utility and validity analysis of the HrQoL measures. For the summary scores, floor and ceiling effects were negligible. In contrast, relevant ceiling effects were observed for single HrQoL domains and the VAS scores. Internal consistency of the 12-item HrQoL questionnaire was above the recommended threshold of 0.7 for Cronbach's alpha needed for comparison between groups. The item-rest correlations were all above 0.25, indicating that single items measured the same construct as the remaining ones and removal of a specific item would not have increased Cronbach's alpha.

**Table 2:** Utility and validity measures of HrQoL instrument from 4,848 school children with complete questionnaire data.

	Scale				
	Domain 1 (physical)	Domain 2 (psychosocial)	Domain 3 (environmental)	Total HrQoL	VAS score
Number of items	5	3	3	12	-
Utility					
Floor (%)^a^	0.5	0.4	0.7	0.0	0.0
Ceiling (%)^a^	22.8	38.0	54.0	2.1	26.2
Internal consistency					
Cronbach α^b^	0.65	0.43	0.48	0.71	-
Relationship with symptom reporting					
Spearman rank sum correlation	−0.28	−0.22	−0.18	−0.37	−0.22
p-value	<0.001*	<0.001*	<0.001*	<0.001*	<0.001*
Association (95% CI)	−1.5 (−1.7, −1.4)	−1.1 (−1.3, −1.0)	−0.9 (−1.0, −0.7)	−1.4 (−1.5, −1.3)	−1.3 (−1.4, −1.1)
p-value	<0.001	<0.001	<0.001	<0.001	<0.001
Relationship with clinical signs					
Association (95% CI)	−1.2 (−1.8, −0.5)	−0.3 (−0.9, 0.3)	0.3 (−0.2, 0.9)	−0.5 (−1.0, −0.1)	−0.5 (−1.2, 0.1)
p-value	0.001*	0.322	0.250	0.012*	0.113

This study was conducted between November 2011 and February 2012 in 92 schools all over Côte d'Ivoire.

a Floor and ceiling correspond to the percentage of scores at the minimum (0) and maximum (100) of the scaling range. Floor or ceiling effects ≤15% are considered acceptable and providing reliable estimates [44].

b All items of the HrQoL instrument added up to the Cronbach α values indicating measurement of the same concept. Values of α≥0.7 are recommended for comparison between groups [56].

To assess the relationship between HrQoL and VAS scores with symptom and disease reporting, a variable providing the total number of self-reported symptoms (n = 11) and diseases (n = 8) for each child was generated first, with a possible range of 0 to 19. Subsequently, Spearman rank correlation and linear regression analysis was performed with instrument scores in relation to the number of self-reported morbidities. All correlations and associations where of negative direction indicating decreasing HrQoL scores for increasing numbers of self-reported symptoms and diseases.

Similarly, a summary variable for 7 examined clinical signs (i.e., anemia, fever, hepatomegaly, splenomegaly, stunting, underweight, and wasting) was generated, with a possible range of 0 to 7, and relationship with HrQoL and VAS scores assessed using linear regression analysis. Particularly the physical health domain showed strong negative association with increased number of clinical signs.

*Statistically significant (p<0.05).

Self-reported symptoms and diseases were reflected in the HrQoL. All HrQoL measures showed significant negative correlations and associations with increasing number of self-reported morbidities. For an incremental increase of 1 self-reported morbidity, the overall HrQoL decreased by 1.4 points (p<0.001). Clinical signs were mainly captured by the physical domain of the HrQoL tool, showing a decreased domain score of 1.2 points (p = 0.001) by each supplemental clinical morbidity observed. VAS scores showed a statistically significant correlation and association with self-reported symptoms and diseases (Table 2) and also a statistically significant correlation with overall HrQoL (all p<0.001), but the correlations were only weak (ρ = −0.22 and ρ = 0.30, respectively). The VAS results were not considered for in-depth analysis and calculation of DWs due to deviance between actual data collected and the original concept of the scale.

### Self-Reported HrQoL

Univariate analysis showed several differences in overall HrQoL among groups with different sociodemographic factors and observed clinical signs (Table 3). Boys reported higher overall HrQoL scores, which were mainly driven by higher self-rated environmental wellbeing. Children from the most poor wealth quintile showed significantly lower scores for all three HrQoL domains. Lower scores for psychosocial and environmental wellbeing, and thus lower overall HrQoL scores, were observed in older children and children living in urban areas. Children from the northern regions reported higher physical but lower environmental wellbeing than their peers from the southern zone. Children's HrQoL with regard to parasitic infections mainly showed differences for the physical domain. Microhematuria negatively affected physical wellbeing, while light-intensity soil-transmitted helminth infections and low *Plasmodium* parasitemia were associated with fewer problems in this domain compared to non-infected counterparts. Comparison for *Plasmodium*-helminth co-infection categories and the number of concurrent parasitic infections (including malaria parasites) showed that children harboring two or more concurrent infections reported the highest physical wellbeing scores compared to their mono- or non-infected counterparts. Anemic children's HrQoL was considerably compromised compared to non-anemic children. A similar but less pronounced decrease in HrQoL was found in children with splenomegaly. Other observed clinical signs showed no significant effects on children's overall HrQoL, but wasted children reported a significantly increased psychosocial wellbeing, while generally malnourished children reported not only higher psychosocial but also higher environmental wellbeing.

**Table 3:** Mean overall and domain HrQoL scores grouped by sociodemographic, parasitological, and clinical variables from 4,848 school children (2,269 females, 2,579 males) in Côte d'Ivoire.

Parameter		Mean scores							
		Total HrQoL	p-value	Domain 1	p-value	Domain 2	p-value	Domain 3	p-value
Sex	Female	76.0		72.9		80.4		84.3	
	Male	77.0	0.004*	74.0	0.071	79.8	0.431	86.4	<0.001*
Age group (years)	5–10	76.7		73.0		80.5		86.5	
	11–16	76.1	0.088	74.5	0.040*	79.0	0.006*	83.0	<0.001*
Socioeconomic status	Most poor	75.1		71.4		79.3		83.9	
	Very poor	77.7		75.6		81.4		85.6	
	Poor	76.0		73.7		79.2		84.4	
	Less poor	76.4		73.4		79.4		86.3	
	Least poor	77.5	<0.001*	73.5	0.003*	81.2	0.020*	86.8	0.030*
Residential area	Rural	76.9		73.6		80.8		86.1	
	Urban	75.1	0.003*	73.2	0.426	77.3	<0.001*	82.9	<0.001*
Ecozone	South	76.5		72.1		80.4		86.4	
	North	76.5	0.429	75.6	<0.001*	79.6	0.220	83.9	<0.001*
*Plasmodium* spp.	Not infected	75.5		71.9		79.4		84.8	
	Infected with <1,000 parasites/µl of blood	77.4		74.8		80.5		85.8	
	Infected with ≥1,000 parasites/µl of blood	75.5	<0.001*	72.2	<0.001*	79.0	0.131	85.1	0.308
*S. haematobium*	No microhematuria	76.7		73.8		80.2		85.4	
	Microhematuria positive	73.5	0.005*	68.9	0.002*	77.9	0.286	84.8	0.245
*S. mansoni*	Not infected	76.4		73.4		80.0		85.3	
	Light-intensity	77.9		75.5		81.0		87.3	
	Moderate- to heavy-intensity	80.4	0.019*	77.9	0.034*	84.2	0.314	87.9	0.584
Soil-transmitted helminths	Not infected	76.2		73.0		79.8		85.4	
	Light-intensity	77.6		75.5		81.1		85.1	
	Moderate- to heavy- intensity	77.9	0.160	74.4	0.019*	80.1	0.605	86.2	0.854
*Plasmodium*-helminth co-infection	Neither of the two infections	75.5		71.9		79.3		84.5	
	*Plasmodium* only	76.7		73.7		80.0		85.8	
	Helminth only	75.8		72.0		79.7		86.2	
	Co-infected	77.2	0.023*	74.8	0.017*	80.9	0.385	85.0	0.127
Number of concurrent parasitic infections	0	75.5		71.9		79.3		84.5	
	1	76.5		73.6		79.9		85.7	
	≥2	77.3	0.013*	74.6	0.015*	81.1	0.211	85.5	0.244
Number of concurrent helminth infections	0	76.3		73.2		79.8		85.5	
	1	77.0		74.2		81.0		84.9	
	≥2	76.8	0.412	74.5	0.291	79.3	0.262	87.4	0.301
Anemia	Not anemic	77.1		74.5		80.7		85.5	
	Anemic	75.0	<0.001*	71.0	<0.001*	78.4	0.001*	85.2	0.413
Wasting	Not wasted	76.3		73.3		79.8		85.2	
	Wasted (Z-score <−2)	77.6	0.120	74.9	0.152	82.1	0.008*	86.4	0.187
Stunting	Not stunted	76.4		73.5		79.9		85.3	
	Stunted (Z-score <−2)	76.8	0.742	73.5	0.862	81.0	0.227	85.8	0.408
Any malnutrition	Not malnourished	76.3		73.5		79.5		85.0	
	Malnourished (Z-score <−2)	77.0	0.326	73.5	0.808	81.5	0.004*	86.4	0.040*
Fever	No fever (<38°C)	76.5		73.5		80.0		85.4	
	Fever (≥38°C)	75.6	0.797	71.2	0.632	83.1	0.273	86.9	0.756
Liver enlargement	Normal	76.5		73.5		80.1		85.4	
	Enlarged	75.8	0.806	73.7	0.853	77,9	0.646	84.4	0.430
Spleen enlargement	Normal	76.7		73.7		80.3		85.4	
	Enlarged (Hackett's scale ≥1)	74.8	0.011*	71.6	0.027*	78.2	0.039*	85.2	0.626

Domain 1 = physical wellbeing; domain 2 = psychosocial wellbeing; domain 3 = environmental wellbeing.

*Statistically significant (p<0.05) based on Wilcoxon rank sum (for variables with 2 categories) and Kruskal-Wallis test (for variables with more than 2 categories).

Table 4 provides an overview on significant associations between sociodemographic, parasitological, and clinical variables on one hand and self-reported HrQoL on the other hand, placing emphasis on summary and physical wellbeing scores, derived from multivariate linear regression with a stepwise backward elimination procedure. Sex, socioeconomic status, anemia, *Plasmodium* spp. infection, *Plasmodium*-helminth co-infection, and number of concurrent parasitic infections remained significant predictors for overall HrQoL. If only physical wellbeing was considered, negative associations of clinical manifestations such as anemia and malnutrition were more pronounced. Interestingly, several single species parasitic infections (i.e., *Plasmodium* spp., and soil-transmitted helminths) and multiple species parasitic infections (i.e., *Plasmodium*-helminth, and number of concurrent infections ≥2) showed a significant positive association with self-reported physical wellbeing.

**Table 4:** Associations between health-related quality of life and physical domain scores with sociodemographic, parasitological, and clinical variables from multivariate regression analysis.

	Health-related quality of life (summary)			Domain 1 (physical wellbeing)^b^		
Variable^a^	Coeff.	95% CI	p-value	Coeff.	95% CI	p-value
Sex (male)	1.0	(0.2, 1.8)	0.015*	1.0	(−0.2, 2.3)	0.113
Age group (11–16 years)	−0.6	(−1.5, 0.2)	0.154	1.6	(0.2, 2.9)	0.025*
Wealth quintile (Most poor)	−1.7	(−2.8, −0.6)	0.002*	−1.7	(−3.3, −0.0)	0.048*
Ecozone (North)	-	-	-	3.7	(0.2, 7.1)	0.036*
*Plasmodium* spp. infected	1.0	(0.0, 2.0)	0.046*	1.6	(0.2, 3.1)	0.029*
Anemia	−1.2	(−2.1, −0.2)	0.013*	−2.0	(−3.4, −0.6)	0.006*
Splenomegaly	−1.1	(−2.4, 0.2)	0.099	-	-	-
Soil-transmitted helminths	-	-	-	1.9	(0.2, 3.5)	0.030*
Any form of malnutrition (Z-score <−2)	-	-	-	−1.5	(−2.9, −0.1)	0.037*
*Plasmodium*-helminth co-infected	1.4	(0.0, 2.7)	0.043*	2.8	(0.7, 4.8)	0.009*
Number of concurrent parasitic infections (≥2)	1.7	(0.4, 3.0)	0.009*	2.9	(0.9, 4.9)	0.004*

Multivariate regression models with random effects to account for clustering and a stepwise backward elimination approach were utilized to identify explanatory variables, which most significantly influence the children's overall quality of life and physical domain scores. Initial models included sociodemographic (e.g., sex, age group, socioeconomic status, residential area (rural or urban), and ecozone), parasitological (by infection intensity for each species investigated), and clinical (anemia, wasting, stunting, fever, hepatomegaly, and splenomegaly) variables. During stepwise removal, variable categories were combined, based on expert knowledge and logical deduction, before eventually eliminating the respective variable. Remaining explanatories were included at a significance level of p<0.2. Quality of life and domain scores were pre-transformed into a scale from 0 to 100, thus coefficients correspond to percentages of change. For variables on concurrent infections (*Plasmodium*-helminth co-infection and number of concurrent parasitic infections), the single parasite variables were exchanged by the concurrent infections variables but the same additional explanatories as for the single species models were used.

CI = confidence interval.

a Reference categories for explanatory variables: sex = female; age group = 5–10 years; wealth quintile = wealthier quintiles (top 80%); ecozone = South; *Plasmodium* = no or low parasitemia (<1,000 parasites/µl of blood); anemia = not anemic; *S. haematobium* = no microhematuria; soil-transmitted helminths = not infected; any form of malnutrition = neither stunted, nor wasted, nor underweight (Z score>−2); *Plasmodium*-helminth co-infected = neither infected with any of the two; number of concurrent infections = not infected with any investigated parasite species.

b Domain 1 showed ceiling effects >15%. Tobit regression models were therefore built additionally for comparison. Except for ecozone and wealth quintile, the same significant relationships were identified in the tobit regression models as in the linear regression analysis presented above.

*Statistically significant (p<0.05).

## Discussion

We present HrQoL measures among 4,848 school-aged children surveyed during a 3-month cross-sectional survey in the dry season in Côte d'Ivoire, and explore associations with parasitic infections and clinical and sociodemographic measures. Parasitological examination revealed a very high prevalence of *Plasmodium* spp. infection (75.0%). Helminth infections were considerably lower; 17.2%, 10.6%, 3.7%, 1.8%, and 1.3% for hookworm, *S. haematobium* (microhematuria), *S. mansoni*, *A. lumbricoides*, and *T. trichiura*, respectively. More than a quarter of the surveyed children showed clinical signs of anemia and malnutrition. Findings from multivariate linear regression analysis revealed that the children's self-rated overall HrQoL and physical wellbeing is lower among those affected by anemia and malnutrition compared to their counterparts without anemia and malnutrition. Surprisingly, associations between HrQoL and parasitic infection status were of positive rather than negative direction. Sociodemographic variables such as sex, age group, socioeconomic status, and setting characteristics had considerable influences on children's perceived HrQoL. The locally adapted HrQoL instrument employed showed acceptable utility considering minimal floor and ceiling effects and a robust internal consistency (Cronbach's α≥0.7). Significant correlations and associations between HrQoL scales and self-reported and clinically assessed morbidity were found and even though the effect sizes were weak, they may further support the concept of health measured by the HrQoL tool.

Interestingly, we could not identify significantly lower HrQoL scores in *Plasmodium*- and helminth-infected children compared to their non-infected peers. Possible explanations for this finding are offered for consideration. First, in Côte d'Ivoire 100% of the population is at risk of *Plasmodium* infection [3] and previous research concluded that malaria transmission is perennial [45], [46]. Constant exposure from early childhood onwards leads to naturally acquired immunity to malaria at an early age [47]. Thus, most of the *Plasmodium* infections we identified in the school-aged population surveyed were asymptomatic (>98%). Levels of transmission and endemicity of parasitic infections has been shown to influence children's HrQoL. For example, Kenyan school-aged children infected with *S. haematobium* from a high endemicity setting reported similar HrQoL measures than their non-infected counterparts, whilst infected children in a low prevalence village reported significantly lower HrQoL compared to non-infected children [23].

Second, our study focused on children who were present at school the day of the survey. Children experiencing a clinical disease episode, perhaps related to a parasite infection, and who might have expressed lowered HrQoL, were more likely to be absent from school than their healthier peers. Helminth infections, additionally, might still be at a less advanced stage with regard to disability in children compared to adolescents or adults. Smaller studies conducted in the People's Republic of China and Kenya also found no evidence of significant differences in self-rated HrQoL between helminth-infected and non-infected school children [24], [26]. Those studies that reported negative associations between HrQoL and helminth infections either focused on adult populations [22] or investigated chronic and advanced clinical stages of an infection [21], [48].

Third, polyparasitism, particularly *Plasmodium*-helminth co-infections, is common in Côte d'Ivoire [49]–[51]. It follows that interactions between multiple species parasitic infections and their influence on ill-health must be considered. Indeed, potentially beneficial effects from light-intensity helminth infections on clinical outcomes (i.e., anemia) and subtle morbidity (i.e., physical fitness) in school-aged populations from malaria co-endemic settings in Côte d'Ivoire have been indicated [46], [50], [51]. Underlying mechanisms might be seen in the immunomodulatory features of helminth infections that down regulate the pro-inflammatory immune response needed to combat intracellular parasites like *Plasmodium*. Consequently, this may negatively affect resistance but simultaneously promote tolerance to malaria-related pathology by controlling harmful associated inflammation [52]. The current study confirms these prior observations, as we observed a positive association between soil-transmitted helminth infections, *Plasmodium*-helminth co-infections, and two or more concurrent infections including malaria parasites, and reported physical wellbeing.

We found significantly lower HrQoL among anemic children compared to non-anemic children. Parasitic infections, most notably *Plasmodium* and hookworm contribute to the development of anemia [53]. *Plasmodium* and helminth mono- or co-infected children in our sample showed significantly higher odds ratios for anemia than their non-infected counterparts (all ORs>1.4). Consequently, we suggest the attribution of direct disease consequences (sequelae) – such as anemia due to specific parasitic infections – to the etiological cause in future burden estimates [14]. We found a 1-point lower HrQoL score overall and a 2-point lower physical wellbeing score on a 100-point scale. If divided by 100, these findings might translate to DWs of 0.01 and 0.02 on the DW scale that ranges from 0 to 1. Such DWs are within the range of recent DW estimates of the GBD 2010 Study, which were set at 0.005, 0.058, and 0.164 for mild, moderate, and severe anemia [15], [54].

The HrQoL concept attempts to evaluate the impact of diseases and injuries from a comprehensive point of view, incorporating psychological, social, and environmental wellbeing on top of physical health [12], [15]. We found that particularly psychosocial and environmental measures of wellbeing were significantly associated with sociodemographic variables like sex, age, socioeconomic status, and residential area. Associations of the physical component of HrQoL with parasite infections and clinical signs were observed to be more pronounced and indicated that the perceived health status varies between and depends importantly on different sociocultural settings. Our results are in line with previous observations [22], [23], [25] and highlight the importance of inclusion of social determinants for more integrative burden of disease assessments.

Our data stem from a large-scale nation-wide survey, which subjected almost 5,000 children to detailed clinical and parasitological examinations, coupled with a questionnaire. A major weakness of previous studies was their small sample sizes [22], [24], [25]. Further, we consider the setting-tailored, applied HrQoL tool as a useful and valid instrument. Its internal consistency was good (Cronbach's α>0.7) and floor and ceiling effects for the overall HrQoL were minimal, despite its shortness, including only 12 items compared to 26 questions in the WHOQOL-Bref, which was used as a template to develop our tool [18]. Particularly the ceiling effects were more pronounced when looking at single domain scores, which is, however, not surprising, considering the lower number of items in each domain. The ceiling effects found for the physical domain, were addressed by utilizing tobit regression analysis, which have been shown to provide more reliable estimates in censored data [55], in parallel to linear regression models. The negative associations and correlations between HrQoL and symptom and disease reporting followed the logic of lower self-rated HrQoL in simultaneously higher experienced morbidity and supported the construct that our instrument measures.

Data collection on a national scale entails several limitations. To respect the tight time schedule and in view of limited financial and human resources, all parasitological, clinical, and questionnaire information had to be collected within a single day at each location by dedicated field teams. Consequently, teachers of the selected schools were trained to administer our questionnaire and they assisted in the conduct of the interview. Given our time constraints and restricted resources, we were not able to assess inter-observer agreement and cannot exclude measurement errors due to variation between interviewers. Another limitation regarding the questionnaire was the difficult implementation of the VAS, as already observed elsewhere [24]. The concept of this scale, the range of 0 to 100, and the fact that children had to point out their respective health status on a sheet was poorly understood. As an adaptation, children were asked to rate and orally express their health status according to a scale they were more familiar with, a scale of school marks (ranging from 0 to 10). However, this procedure resulted in a categorical rather than an interval distribution of scores. Unfortunately, this limitations hindered us to fully exploit these data and derive DWs, which could have been compared with previous research conducted elsewhere focusing on chronic *S. japonicum* infection [48]. Hence, there is a pressing need for a culturally accepted alternative to the VAS.

We conclude that the assessment of HrQoL in school-aged children in areas where parasitic infections are still widespread tends to be difficult and may not be sensitive enough to capture subtle morbidities. Important factors blurring the picture might be the often asymptomatic course due to acquired immunity in malaria and more subtle morbidities at this age for helminth infections, which therefore may not be perceived as disabling by infected children. School absenteeism adds bias, as non-inclusion of children who might experience more measurable disability will not be part of the analysis. Importantly though, the applied instrument showed acceptable utility and validity and was able to identify significant disability of more chronic sequelae such as anemia. Further refinement and more rigorous reliability measurements of the tool are needed. Surveys in settings targeting specific parasite endemicity levels and efforts to include non-enrolled and otherwise absent school-aged children might resolve some of the limitations highlighted here. The aim of developing, validating, and applying setting-specific HrQoL tools that will allow comparison between areas and measuring changes over time remains – particularly as large-scale control efforts targeting malaria and the NTDs are underway.

## Supporting Information

Table S1 **Prevalence and intensity of parasitic infections, stratified by sex, age group, residential area, and ecozone.**
(DOCX)

Table S2 **Clinical signs and self-reported symptoms and diseases, stratified by sex, age group, residential area, and ecozone.**
(DOCX)

Data set S1 **Data set.**
(XLSX)

Appendix S1 **Questionnaire for HrQoL assessment and VAS (in French).**
(DOC)

Checklist S1 **STROBE checklist.**
(DOC)
