# Supplementary material for: Health-Related Quality of Life among School Children with Parasitic Infections: Findings from a National Cross-Sectional Survey in Côte d'Ivoire
Source: PLoS Negl Trop Dis. 2014 Dec 4;8(12):e3287. doi: 10.1371/journal.pntd.0003287 (PMC4256278; doi:10.1371/journal.pntd.0003287)
Supplement: Table S1 — Prevalence and intensity of parasitic infections, stratified by sex, age group, residential area, and ecozone. (DOCX) [file pntd.0003287.s001.docx]

**Table S1. Prevalence and intensity of parasitic infections, stratified by sex, age group, residential area, and ecozone among 4,848 school children in Côte d'Ivoire.**

| **Parasitic infection** | | **Total** |  | **Age group (years)** | | |  | **Sex** | | |  | **Residential area** | | |  | **Ecozone** | | |
| --- | --- | --- | --- | --- | --- | --- | --- | --- | --- | --- | --- | --- | --- | --- | --- | --- | --- | --- |
|  |  |  |  | 5-10 | 11-16 | p-value |  | Females | Males | p-value |  | Rural | Urban | p-value |  | South | North | p-value |
|  |  | (n=4,848) |  | (n=3,282) | (n=1,566) |  |  | (n=2,269) | (n=2,579) |  |  | (n=3,783) | (n=1,065) |  |  | (n=2,862) | (n=1,986) |  |
| *P. falciparum* | | 74.1 |  | 73.6 | 75.1 | 0.281 |  | 71.6 | 76.4 | <0.001* |  | 77.1 | 63.4 | <0.001* |  | 70.7 | 79.0 | <0.001* |
| *P. malariae* | | 3.9 |  | 4.5 | 2.8 | 0.004* |  | 3.8 | 4.1 | 0.560 |  | 4.4 | 2.4 | 0.003* |  | 3.8 | 4.1 | 0.531 |
| *P. ovale* | | 0.3 |  | 0.3 | 0.2 | 0.476 |  | 0.3 | 0.3 | 0.963 |  | 0.3 | 0.1 | 0.213 |  | 0.4 | 0.1 | 0.060 |
| *Plasmodium* spp. | | 75.0 |  | 74.5 | 75.9 | 0.293 |  | 72.2 | 77.4 | <0.001* |  | 78.1 | 63.9 | <0.001* |  | 71.8 | 79.6 | <0.001* |
|  | Parasitemia ≥1,000 parasites/µl of blood | 23.4 |  | 24.9 | 20.2 | <0.001* |  | 21.8 | 24.8 | 0.015 |  | 24.0 | 21.4 | 0.084 |  | 23.4 | 23.4 | 0.970 |
| *S. haematobium* | | 5.7 |  | 4.7 | 7.7 | <0.001* |  | 5.4 | 5.9 | 0.443 |  | 5.7 | 5.7 | 0.956 |  | 7.6 | 3.0 | <0.001* |
| *S. mansoni* | | 3.7 |  | 2.7 | 5.7 | <0.001* |  | 1.9 | 5.2 | <0.001* |  | 3.4 | 4.4 | 0.133 |  | 4.2 | 2.9 | 0.016* |
|  | Light infection^a^ | 48 |  | 54.6 | 41.6 |  |  | 47.7 | 48.1 |  |  | 44.6 | 57.5 |  |  | 45.0 | 54.4 |  |
|  | Moderate infection^a^ | 33.9 |  | 25.0 | 42.7 |  |  | 29.6 | 35.3 |  |  | 35.4 | 29.8 |  |  | 34.2 | 33.3 |  |
|  | Heavy infection^a^ | 18.1 |  | 20.5 | 15.7 | 0.045 |  | 22.7 | 16.5 | 0.598 |  | 20.0 | 12.8 | 0.286 |  | 20.8 | 12.3 | 0.320 |
| Hookworm | | 17.2 |  | 13.7 | 24.6 | <0.001* |  | 11.1 | 22.6 | <0.001* |  | 20.0 | 7.4 | <0.001* |  | 16.5 | 18.3 | 0.090 |
|  | Light infection^a^ | 96.8 |  | 97.6 | 95.8 |  |  | 97.6 | 96.4 |  |  | 96.6 | 98.7 |  |  | 96.0 | 97.8 |  |
|  | Moderate infection^a^ | 1.9 |  | 1.1 | 2.9 |  |  | 0.8 | 2.4 |  |  | 2.0 | 1.3 |  |  | 1.9 | 1.9 |  |
|  | Heavy infection^a^ | 1.3 |  | 1.3 | 1.3 | 0.186 |  | 1.6 | 1.2 | 0.273 |  | 1.5 | 0.0 | 0.502 |  | 2.1 | 0.3 | 0.067 |
| *A. lumbricoides* | | 1.8 |  | 1.5 | 2.6 | 0.010* |  | 1.8 | 1.9 | 0.723 |  | 2.1 | 0.9 | 0.006* |  | 2.8 | 0.5 | <0.001* |
|  | Light infection^a^ | 84.3 |  | 83.7 | 85.0 |  |  | 85.0 | 83.7 |  |  | 83.8 | 88.9 |  |  | 83.8 | 88.9 |  |
|  | Moderate infection^a^ | 15.7 |  | 16.3 | 15.0 |  |  | 15.0 | 16.3 |  |  | 16.3 | 11.1 |  |  | 16.3 | 11.1 |  |
|  | Heavy infection^a^ | 0.0 |  | 0.0 | 0.0 | 0.864 |  | 0.0 | 0.0 | 0.864 |  | 0.0 | 0.0 | 0.688 |  | 0.0 | 0.0 | 0.688 |
| *T. trichiura* | | 1.3 |  | 1.3 | 1.2 | 0.639 |  | 1.2 | 1.3 | 0.689 |  | 1.2 | 1.5 | 0.418 |  | 1.6 | 0.8 | 0.019 |
|  | Light infection^a^ | 100.0 |  | 100.0 | 100.0 |  |  | 100.0 | 100.0 |  |  | 100.0 | 100.0 |  |  | 100.0 | 100.0 |  |
|  | Moderate infection^a^ | 0.0 |  | 0.0 | 0.0 |  |  | 0.0 | 0.0 |  |  | 0.0 | 0.0 |  |  | 0.0 | 0.0 |  |
|  | Heavy infection^a^ | 0.0 |  | 0.0 | 0.0 | 1.000 |  | 0.0 | 0.0 | 1.000 |  | 0.0 | 0.0 | 1.000 |  | 0.0 | 0.0 | 1.000 |
| Soil-transmitted helminths | | 19.1 |  | 15.5 | 26.8 | <0.001* |  | 13.3 | 24.2 | <0.001* |  | 21.9 | 9.1 | <0.001* |  | 19.0 | 19.2 | 0.843 |
|  | Light infection^a^ | 95.6 |  | 96.3 | 94.8 |  |  | 96 | 95.4 |  |  | 95.3 | 97.9 |  |  | 94.1 | 97.6 |  |
|  | Moderate infection^a^ | 3.2 |  | 2.6 |  | 4.1 |  | 2.7 | 3.5 |  |  | 3.4 | 2.1 |  |  | 4.0 | 2.1 |  |
|  | Heavy infection^a^ | 1.2 |  | 1.2 |  | 1.2 |  | 1.3 | 1.1 | 0.760 |  | 1.3 | 0.0 | 0.404 |  | 1.8 | 0.3 | 0.022* |

Parasite prevalences are provided in % of all included school children. Data on infection intensities are provided as % of all positive cases.

^a^Intensities of intestinal helminth infections are categorized according to WHO guidelines [7].

*Statistically significant (p<0.05) based on χ^2^statistics.
