# Supplementary material for: Health-Related Quality of Life among School Children with Parasitic Infections: Findings from a National Cross-Sectional Survey in Côte d'Ivoire
Source: PLoS Negl Trop Dis. 2014 Dec 4;8(12):e3287. doi: 10.1371/journal.pntd.0003287 (PMC4256278; doi:10.1371/journal.pntd.0003287)
Supplement: Table S2 — Clinical signs and self-reported symptoms and diseases, stratified by sex, age group, residential area, and ecozone. (DOCX) [file pntd.0003287.s002.docx]

**Table S2. Clinical signs and self-reported symptoms and diseases, stratified by sex, age group, residential area, and ecozone among 4,848 school children in Côte d'Ivoire.**

| **Morbidity** | | **Total** |  | **Age group (years)** | | |  | **Sex** | | |  | **Residential area** | | |  | **Ecozone** | | |
| --- | --- | --- | --- | --- | --- | --- | --- | --- | --- | --- | --- | --- | --- | --- | --- | --- | --- | --- |
|  |  |  |  | 5-10 | 11-16 | p-value |  | Females | Males | p-value |  | Rural | Urban | p-value |  | South | North | p-value |
|  |  | (n=4,848) |  | (n=3,282) | (n=1,566) |  |  | (n=2,269) | (n=2,579) |  |  | (n=3,783) | (n=1,065) |  |  | (n=2,862) | (n=1,986) |  |
| Observed clinical signs | |  |  |  |  |  |  |  |  |  |  |  |  |  |  |  |  |  |
|  | Mean hemoglobin level (g/l) | 121.8 |  | 121.1 | 123.3 | <0.001* |  | 122.7 | 121.1 | <0.001* |  | 121.9 | 121.7 | 0.796 |  | 119.9 | 124.6 | <0.001* |
|  | Anemia^a^ | 28.7 |  | 27.3 | 31.6 | 0.002* |  | 25.9 | 31.2 | <0.001* |  | 29.2 | 27.0 | 0.178 |  | 33.4 | 21.9 | <0.001* |
|  | Wasting^b^ | 11.8 |  | 10.2 | 15.3 | <0.001* |  | 10.3 | 13.2 | 0.001* |  | 12.5 | 9.6 | 0.010* |  | 11.9 | 11.8 | 0.918 |
|  | Stunting^b^ | 18.1 |  | 12.6 | 29.4 | <0.001* |  | 15.5 | 20.3 | <0.001* |  | 18.4 | 16.8 | 0.233 |  | 17.6 | 18.7 | 0.303 |
|  | Any form of malnutrition^c^ | 28.4 |  | 23.4 | 38.8 | <0.001* |  | 24.5 | 31.8 | <0.001* |  | 29.1 | 25.7 | 0.031* |  | 28.3 | 28.5 | 0.860 |
|  | Liver enlargement^d^ | 2.6 |  | 2.7 | 2.5 | 0.743 |  | 1.7 | 3.4 | <0.001* |  | 2.5 | 3.1 | 0.246 |  | 2.6 | 2.7 | 0.800 |
|  | Spleen enlargement^d^ | 11.5 |  | 12.3 | 9.9 | 0.014* |  | 10.7 | 12.3 | 0.093 |  | 12.4 | 8.5 | 0.001* |  | 9.3 | 14.7 | <0.001* |
|  | Fever (≥38 °C) | 1.9 |  | 1.9 | 1.9 | 0.987 |  | 2.2 | 1.6 | 0.142 |  | 1.8 | 2.0 | 0.752 |  | 2.0 | 1.6 | 0.292 |
|  | Clinical malaria^e^ | 1.4 |  | 1.4 | 1.4 | 0.940 |  | 1.5 | 1.3 | 0.511 |  | 1.5 | 1.0 | 0.223 |  | 1.6 | 1.2 | 0.194 |
| Self-reported symptoms | |  |  |  |  |  |  |  |  |  |  |  |  |  |  |  |  |  |
|  | Headache | 54.3 |  | 52.4 | 58.4 | <0.001* |  | 59 | 50.2 | <0.001* |  | 54.6 | 53.3 | 0.468 |  | 50.5 | 59.8 | <0.001* |
|  | Abdominal pain | 51.1 |  | 49.1 | 55.2 | <0.001* |  | 55.6 | 47.2 | <0.001* |  | 50.7 | 52.5 | 0.303 |  | 47 | 57 | <0.001* |
|  | Fatigue | 48.6 |  | 46 | 54 | <0.001* |  | 51 | 46.5 | 0.002* |  | 48.3 | 49.6 | 0.469 |  | 46.1 | 52.2 | <0.001* |
|  | Fever | 48.2 |  | 46.7 | 51.2 | 0.003 |  | 53 | 43.9 | <0.001* |  | 47.9 | 49.1 | 0.485 |  | 42.3 | 56.7 | <0.001* |
|  | Vomiting/nausea | 35 |  | 34.7 | 35.5 | 0.599 |  | 36.7 | 33.5 | 0.021* |  | 35 | 34.8 | 0.909 |  | 30.6 | 41.3 | <0.001* |
|  | Diarrhea | 31.5 |  | 30.6 | 33.3 | 0.06 |  | 32.5 | 30.5 | 0.133 |  | 31.2 | 32.3 | 0.502 |  | 28.9 | 35.2 | <0.001* |
|  | Blood in stool | 30 |  | 28.6 | 32.7 | 0.004* |  | 31.2 | 28.9 | 0.074 |  | 30.4 | 28.4 | 0.199 |  | 29.1 | 31.2 | 0.123 |
|  | Loss of appetite | 28.9 |  | 27.5 | 31.8 | 0.002* |  | 30.9 | 27.1 | 0.004* |  | 27.6 | 33.3 | <0.001 |  | 26.7 | 31.9 | <0.001* |
|  | Respiratory problems | 26.8 |  | 26 | 28.6 | 0.054 |  | 28.9 | 25.1 | 0.003* |  | 27.7 | 23.7 | 0.008* |  | 23.8 | 31.2 | <0.001* |
|  | Dysentery | 24.1 |  | 23.6 | 25.3 | 0.195 |  | 26.2 | 22.3 | 0.002* |  | 24.5 | 23 | 0.33 |  | 23.2 | 25.5 | 0.059 |
|  | Blood in urine | 10.1 |  | 11.2 | 9.6 | 0.077 |  | 9.7 | 10.5 | 0.35 |  | 10.6 | 8.6 | 0.068 |  | 11.3 | 8.5 | 0.002* |
| Self-reported diseases | |  |  |  |  |  |  |  |  |  |  |  |  |  |  |  |  |  |
|  | Cough | 57.3 |  | 57.7 | 56.3 | 0.351 |  | 58.4 | 56.3 | 0.126 |  | 57.5 | 56.5 | 0.573 |  | 53.2 | 63.1 | <0.001* |
|  | Cold | 46.1 |  | 46.6 | 45.3 | 0.402 |  | 48 | 44.5 | 0.013* |  | 46.2 | 45.8 | 0.812 |  | 40.3 | 54.6 | <0.001* |
|  | Malaria | 30.4 |  | 29.3 | 32.5 | 0.025* |  | 32.4 | 28.6 | 0.004* |  | 30.6 | 29.7 | 0.578 |  | 24.6 | 38.6 | <0.001* |
|  | Malnutrition | 21.4 |  | 21.2 | 21.9 | 0.564 |  | 20.9 | 21.9 | 0.407 |  | 21.5 | 21.2 | 0.864 |  | 17.9 | 26.5 | <0.001* |
|  | Eye disease | 19.1 |  | 18.7 | 20 | 0.301 |  | 20.8 | 17.7 | 0.007* |  | 18.5 | 21.6 | 0.021* |  | 17.5 | 21.5 | 0.001* |
|  | Worms | 16.8 |  | 16.4 | 17.5 | 0.336 |  | 17.4 | 16.2 | 0.282 |  | 17.2 | 15 | 0.088 |  | 15.3 | 18.8 | 0.002* |
|  | Schistosomiasis | 14.2 |  | 13 | 16.5 | 0.001* |  | 14.5 | 13.9 | 0.567 |  | 13.9 | 15.1 | 0.305 |  | 14.9 | 13 | 0.065 |
|  | Skin disease | 13.1 |  | 12.9 | 13.6 | 0.473 |  | 11.7 | 14.4 | 0.006* |  | 13 | 13.4 | 0.719 |  | 13.3 | 12.8 | 0.596 |
| Mean no. of reported morbidities | | 6.1 |  | 5.9 | 6.4 | <0.001* |  | 6.4 | 5.8 | <0.001* |  | 6.1 | 6.1 | 0.985 |  | 5.6 | 6.8 | <0.001* |

Prevalences of clinical or self-reported morbidities are provided in % of all included school children with the exception of hemoglobin levels and the number of self-reported morbidities, where mean values are displayed.

^a^Defined as hemoglobin levels below 115 g/l and below 120 g/l in children aged 5-11 years and 12-16 years, respectively.

^b^Calculated according to WHO child growth standards [42]; defined as BMI-for-age (wasting) and height-for-age (stunting) resulting in a Z-score < -2.

^c^Defined as any of the assessed nutritional indicators resulting in a Z-score < -2; this includes wasting, stunting, and weight-for-age (underweight).

^d^Defined as palpable liver and spleen (≥grade I by Hackett’s classification), respectively.

^e^Clinical malaria is defined as being *Plasmodium* positive and having fever (≥38° C)

*Statistically significant (p<0.05) based on t-test (Hb, mean no. of reported morbidities) and chi-square (prevalence of specific clinical and self-reported morbidities) statistics
