# Supplementary material for: Health-Related Quality of Life among School Children with Parasitic Infections: Findings from a National Cross-Sectional Survey in Côte d'Ivoire
Source: PLoS Negl Trop Dis. 2014 Dec 4;8(12):e3287. doi: 10.1371/journal.pntd.0003287 (PMC4256278; doi:10.1371/journal.pntd.0003287)

#### **Questionnaire en milieu scolaire Code de l’école………………………..**

| Localité:…………………………………………… Classe : CP2 CE1 CE2 CM1 CM2  École:…………………………………………... Code de la classe : 0 1 2 3 4 |
| --- |

Nom et signature de l’enquêteur :……………………………………………………Date :………………

| **Elève N°** | | **01** | **02** | **03** | **04** | **05** | **06** | **07** | **08** | **09** | **10** | **11** | **12** | **13** | **14** | **15** | **16** | **17** | **18** | **19** | **20** | **21** | **22** | **23** | **24** | **25** |
| --- | --- | --- | --- | --- | --- | --- | --- | --- | --- | --- | --- | --- | --- | --- | --- | --- | --- | --- | --- | --- | --- | --- | --- | --- | --- | --- |
| **5. Qualité de vie** | | | | | | | | | | | | | | | | | | | | | | | | | | |
| 1) | En général, comment tu te sens? Dis-tu ta santé est : (très faible=1, faible=2, ni f ni b=3, bonne=4, très bonne=5) |  |  |  |  |  |  |  |  |  |  |  |  |  |  |  |  |  |  |  |  |  |  |  |  |  |
| 2) | As-tu des problèmes pour marcher sur une distance très longue (comme marcher jusqu’ au prochain village ou marcher de la maison jusqu’aux champs plus loin)? (Oui b.d.p.=1, Oui q.p.=2, Non p.d.p.=3) |  |  |  |  |  |  |  |  |  |  |  |  |  |  |  |  |  |  |  |  |  |  |  |  |  |
| 3) | As-tu des problèmes pour monter sur une côte ? (Oui b.d.p.=1, Oui q.p.=2, Non p.d.p.=3) |  |  |  |  |  |  |  |  |  |  |  |  |  |  |  |  |  |  |  |  |  |  |  |  |  |
| 4) | As-tu assez de force pour porter l’eau du marigot/puit à la maison ? (🡪filles)  As-tu assez de force pour porter le fargot du champ à la maison? (🡪garçons) (Non p.d.t.=1, Parfois oui p. non=2, Oui toujours=3) |  |  |  |  |  |  |  |  |  |  |  |  |  |  |  |  |  |  |  |  |  |  |  |  |  |
| 5) | As-tu des problèmes pour te laver ou t’habiller tout(e) seul(e)? (Oui b.d.p.=1, Oui q.p.=2, Non p.d.p.=3) |  |  |  |  |  |  |  |  |  |  |  |  |  |  |  |  |  |  |  |  |  |  |  |  |  |
| 6) | As-tu des douleurs ou gêne qui t’empêche de faire ce dont tu as envie ? (Oui b.d.d/g=1, Oui q.d/g=2, Non p.d.d/g=3) |  |  |  |  |  |  |  |  |  |  |  |  |  |  |  |  |  |  |  |  |  |  |  |  |  |
| 7) | As-tu des problèmes pour être attentif (ve)/ te concentrer (par exemple à l’école ou faisant tes devoirs) ? (Oui b.d.p.=1, Oui q.p.=2, Non p.d.p.=3) |  |  |  |  |  |  |  |  |  |  |  |  |  |  |  |  |  |  |  |  |  |  |  |  |  |
| 8) | As-tu souvent des soucis ou es-tu triste ou malheureux (se)? (Oui tout le temps=1, Oui parfois=2, Non jamais=3) |  |  |  |  |  |  |  |  |  |  |  |  |  |  |  |  |  |  |  |  |  |  |  |  |  |
| 9) | Parles-tu facilement avec ton papa ou ta maman? (Npn,P.d.t..=1, Oui, quelques fois=2, Oui, facilement=3) |  |  |  |  |  |  |  |  |  |  |  |  |  |  |  |  |  |  |  |  |  |  |  |  |  |
| 10) | As-tu assez de temps pour t’amuser/jouer avec tes camerades ? (Non, p.d.t.=1, Oui, quelques fois=2, Oui, toujours=3) |  |  |  |  |  |  |  |  |  |  |  |  |  |  |  |  |  |  |  |  |  |  |  |  |  |
| 11) | Te sens-tu en sécurité à la maison ou dans le village où tu habites ? (Non jamais=1, Oui parfois=2, Oui toujours=3) |  |  |  |  |  |  |  |  |  |  |  |  |  |  |  |  |  |  |  |  |  |  |  |  |  |
| 12) | Aimes-tu l’endroit où tu habites ? (Non pas du tout=1, Oui un peu=2, Oui beaucoup=3) |  |  |  |  |  |  |  |  |  |  |  |  |  |  |  |  |  |  |  |  |  |  |  |  |  |
| VAS score (valeur de l’échelle) | |  |  |  |  |  |  |  |  |  |  |  |  |  |  |  |  |  |  |  |  |  |  |  |  |  |


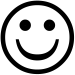


90

80

70

60

50

40

30

20

10

100

**Pire état de santé imaginable**

0

**Meilleur état de**

**santé imaginable**

**Quel est ton état de santé AUJOURD’HUI?**

Nous aimerions savoir si ton état de santé est bon AUJOURD’HUI

L’échelle est graduée de 0 à 10 .

L’échelle représentent les differents notes de santé sur dix. Commes tes differentes notes des devoirs de mathématique par example sur dix.

10 signifie le meilleur état de santé imaginable.

0 signifie le pire état de santé imaginable.

Quelle note sur dix de santé te donnes-tu aujourd’hui?


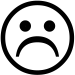

Supplement: Appendix S1 — Questionnaire for HrQoL assessment and VAS (in French). (DOC) [file pntd.0003287.s004.doc]
